# Twelve weeks of protracted venous infusion of fluorouracil (5-FU) is as effective as 6 months of bolus 5-FU and folinic acid as adjuvant treatment in colorectal cancer

**DOI:** 10.1038/sj.bjc.6600995

**Published:** 2003-06-10

**Authors:** A Saini, A R Norman, D Cunningham, I Chau, M Hill, D Tait, T Hickish, T Iveson, F Lofts, D Jodrell, P J Ross, J Oates

**Affiliations:** 1 Royal Marsden Hospital, London and Surrey, UK; 2 Mid Kent Oncology Centre, Maidstone, UK; 3 Royal Bournemouth and Poole Hospitals, Dorset, UK; 4 Royal South Hants Hospital, Southampton, UK; 5 St George's Hospital, London, UK; 6 Western General Hospital, Edinburgh, UK

**Keywords:** protracted intravenous infusion, fluorouracil, folinic acid; adjuvant therapy, colorectal cancer

## Abstract

We performed a multicentre randomised trial to compare the efficacy and toxicity of 12 weeks of 5-fluorouracil (5-FU) delivered by protracted intravenous infusion (PVI 5-FU) against the standard bolus regimen of 5-FU and folinic acid (5-FU/FA) given for 6 months as adjuvant treatment in colorectal cancer. A total of 716 patients with curatively resected Dukes' B or C colorectal cancer were randomised to 5-FU/FA (5-FU 425 mg m^−2^ i.v. and FA 20 mg m^−2^ i.v. bolus days 1–5 every 28 days for 6 months) or to PVI 5-FU alone (300  mg m^−2^ day for 12 weeks). With a median follow-up of 19.8 months, 133 relapses and 77 deaths have been observed. Overall survival did not differ significantly (log rank *P*=0.764) between patients receiving 5-FU/FA and PVI 5-FU (3-year survival 83.2 *vs* 87.9%, respectively). Patients in the 5-FU/FA group had significantly worse relapse-free survival (RFS, log rank *P*=0.023) compared to those receiving PVI 5-FU (3-year RFS, 68.6 *vs* 80%, respectively). Grades 3–4 neutropenia, diarrhoea, stomatitis and severe alopecia were significantly less (*P*<0.0001) and global quality of life scores significantly better (*P*<0.001) for patients in the PVI 5-FU treatment arm. In conclusion, infused 5-FU given over 12 weeks resulted in similar survival to bolus 5-FU and FA over a 6 month period, but with significantly less toxicity.

Approximately 1 million new cases of colorectal cancers were diagnosed worldwide per annum with 500 000 patients dying from the disease in 2000 (Ferlay *et al*, 2001). Despite potentially curative surgery, nearly 40% of patients still experience disease relapse leading to significant morbidity or eventually mortality.

A number of randomised clinical trials performed over the last two decades have established the role of adjuvant therapy in Dukes'C (stage III) node-positive colon cancer. The mature results from the US Intergroup 0035 study showed a 40% reduction in recurrence and 33% reduction in mortality (Moertel *et al*, 1990, 1995). This led a National Institute of Health Consensus Conference to recommend 5-fluorouracil (5-FU)/levamisole to be given for 12 months as adjuvant therapy in patients with stage III colon cancer (NIH Consensus Conference, 1990). The efficacy of 5-FU and folinic acid (FA) as adjuvant therapy was confirmed in the Intergroup-0085, NSABP C-03 and IMPACT-1 studies (Wolmark *et al*, 1993; International Multicentre Pooled Analysis of Colon Cancer Trials (IMPACT) investigators, 1995; O'Connell *et al*, 1997). Furthermore, there appeared to be no detrimental effect on survival in reducing treatment duration from 12 to 6 months (O'Connell *et al*, 1998). The role of adjuvant therapy in Dukes' B (stage II) colon cancer remains controversial with conflicting evidence and no international consensus (International Multicentre Pooled Analysis of B2 Colon Cancer Trials (IMPACT B2) Investigators, 1999; Mamounas *et al*, 1999; Taal *et al*, 2001).

Adjuvant chemotherapy has also been used in Dukes' B and C rectal cancer and has been shown to improve survival compared to surgery alone (Fisher *et al*, 1988), and the efficacy of adjuvant 5-FU/FA in rectal cancer has also been demonstrated (Wolmark *et al*, 2000; Tepper *et al*, 2002). Although a significant reduction in local recurrence is observed, postoperative radiotherapy has not impacted on survival – an observation confirmed in a recent meta-analysis (Colorectal Cancer Collarborative Group, 2001). Radiotherapy (RT) is often used in conjunction with chemotherapy in rectal cancer. Whereas postoperative chemoradiation has been shown to produce superior survival compared to surgery (Gastrointestinal Tumor Study Group, 1985; Douglass Jr *et al*, 1986) or radiotherapy alone (Krook *et al*, 1991), no survival advantage has been demonstrated compared to chemotherapy alone (Gastrointestinal Tumor Study Group, 1985; Wolmark *et al*, 2000).

Two bolus 5-FU/FA dose schedules – Mayo clinic regimen (5-FU 425 mg m^−2^ and FA 20 mg m^−2^ days 1–5 every 4 weeks for six cycles) or Roswell Park Regimen (5-FU 500 mg m^−2^ and FA 500 mg m^−2^ weekly × 6 every 8 weeks for three to four cycles) – have been widely adopted as the reference adjuvant therapy for colon cancer. Protracted intravenous infusion of 5-FU (PVI 5-FU) has been shown to result in higher response rate and lower haematological toxicity compared to bolus injection in patients with advanced colorectal cancer (Lokich *et al*, 1989). In a meta-analysis of six randomised studies, prolonged infusion of 5-FU resulted in less haematological toxicity and a small but statistically significant survival advantage over bolus regimen (Meta-analysis Group In Cancer, 1998), thus providing the rationale to investigate infused 5-FU as adjuvant therapy. Furthermore, the use of PVI 5-FU during radiation has been shown to improve survival compared to bolus 5-FU in rectal cancer (O'Connell *et al*, 1994). In this prospective randomised study, we compared the efficacy of PVI 5-FU against bolus 5-FU/FA as adjuvant therapy in patients with potentially curative resected colorectal cancer. The duration of chemotherapy in the PVI-5-FU arm was shortened to 12 weeks since this still has twice the total intended 5-FU dose delivery compared to bolus schedule (25.2 *vs* 12.75 g m^−2^, respectively). Moreover, in published literature, the greatest improvement in survival was noted in studies in which the largest dose of 5-FU was planned, particularly over the first 3 months (Zalcberg *et al*, 1996).

## PATIENTS AND METHODS

### Patient eligibility

Patients were entered into the study within 12 weeks of curative resection of Dukes' B or C adenocarcinomas of the colon or rectum, providing there was no evidence of metastatic disease as assessed by postoperative radiological and carcinoembryonic antigen (CEA) evaluation. Surgical specimens or representative slides were reviewed in the histopathology department at the participating centres to confirm tumour stage and resection margin status. Circumferential resection margins were required to be clear by at least 1 mm in patients with rectal cancer. Tumours were classified as rectal if they arose below the peritoneal reflection or were within 12 cm of the anal verge. Patients with histopathologically confirmed tumour extension into adjacent organs were eligible if all tumour had been removed and the resection margins were confirmed as being clear. Patients were required to have adequate bone marrow, renal and liver function and no concurrent severe or life-threatening illness. Preoperative RT was allowed in patients with rectal cancer.

Participating patients gave written informed consent before they entered the study. The protocol was approved by the Scientific and Research Ethics Committee of the institutions taking part as well as the North Thames Multi-centre Research Ethics Committee.

### Randomisation procedure

Details of all eligible patients were forwarded to the data manager's office based at the Royal Marsden Hospital, Surrey, UK to verify eligibility criteria. Patients were then randomly assigned by an independent randomisation office to either bolus 5-FU/FA or PVI 5-FU in a 1 : 1 basis using random permuted blocks. Randomisation was stratified by treatment centre and in cases of rectal cancer, whether preoperative RT was given.

### Chemotherapy

Patients were randomly allocated to one of the following treatments:

1. *5-fluorouracil alone by protracted venous infusion (PVI 5-FU).* This was administered via a skin-tunnelled central venous catheter placed in the subclavian vein (Hickman line). Warfarin (1 mg day^−1^) was administered to prevent catheter thrombosis. 5-fluorouracil was given as a continuous intravenous infusion at a dose of 300 mg m^−2^ day^−1^ using a portable pump for 12 weeks.

2. *Intermittent bolus 5-FU and folinic acid (5-FU/FA).* Folinic acid at a dose of 20 mg m^−2^ per day was administered as a bolus intravenous injection followed by a bolus injection of 5-FU at a dose of 425 mg m^−2^ on five consecutive days, repeated every 28 days for a total of 6 cycles. In 1998, the protocol was amended so that the starting dose of bolus 5-FU was reduced to 370 mg m^−2^ for patients aged over 70 years.

### Radiotherapy

In this study, patients with rectal cancer were not routinely treated with combined modality therapy. The addition of adjuvant radiotherapy was specifically reserved for those patients at high risk of locoregional failure (T4 tumours). Post-operative radiotherapy was delivered as 45 Gy in 25 fractions to the pelvis with a 5.4 Gy boost in three fractions to the tumour bed. Irradiation was initiated to coincide with the 4th cycle of bolus therapy or after completion of 12 weeks of PVI 5-FU, which continued at a reduced dose of 200 mg m^−2^ until completion of radiotherapy. For patients in the 5-FU/FA arm, the dose modification was to reduce 5-FU by 75 mg m^−2^ day^−1^ and to truncate the duration to 4 days commencing at the 4th cycle. Cycle 5 was delivered during radiotherapy and cycle 6 was delivered following completion of radiotherapy.

### Toxicity assessment and dose modifications

Adverse effects were graded according to National Cancer Institute Common Toxicity Criteria. Nonhaematological grade 1 toxicities were treated as follows: diarrhoea-codeine phosphate 30–60 mg p.o. qds, stomatitis-sucralfate 1 g qds, and plantar-palmar erythema-pyridoxine 50 mg p.o. tds. If symptoms did not improve with these measures or toxicities were greater than grade 2 at onset, the 5-FU infusion was suspended until toxicity resolved (usually 7–10 days). In the PVI 5-FU arm, dose reductions of 50, 100 and 150 mg m^−2^ were made for grades 2, 3 and 4 toxicities, respectively. In the 5-FU/FA arm, 5-FU dose reductions of 25, 50 and 50% of full dose were made for grades 2, 3 and 4 nonhaematological toxicities, respectively. In addition, if white blood count was <3.0 × 10^9^ l^−1^, absolute neutrophil count was <1.5 × 10^9^ l^−1^ and platelet count was <100 × 10^9^ l^−1^ on the day of treatment, both 5-FU and FA would be withheld for 1 week. If dose delay for 2 weeks was required, all subsequent 5-FU treatment would be given at 75% of full dose. If delay of >2 weeks was required, all subsequent 5-FU treatment would be given at 50% of full dose. No dose reductions were made to FA for toxicity.

### Quality of life (QOL) assessment

The multidimensional health-functioning questionnaire from the European Organisation for Research and Treatment of Cancer (EORTC QLQ-C30) was used to assess QOL of patients undergoing chemotherapy. Scoring was made according to the guidelines provided by the EORTC QOL group, using standardised procedures. Scores were measured weekly in the first month of treatment and then at monthly intervals.

### Follow-up assessment

Following completion of chemotherapy, patients were seen every 3 months until 1 year from the start of chemotherapy and then six-monthly. Carcinoembryonic antigen was estimated at each clinical visit. Re-evaluation CT scans were performed 12 and 24 months after the start of chemotherapy. Colonoscopy was performed 12 months after the start of chemotherapy.

### Statistical methods

The accrual goal was set at 716 patients, which was calculated to detect a minimal improvement in patient survival from 60 to 70% after 5 years of follow up (358 patients per arm to provide at least 80% power in a two-sided test *α*=5%). The primary end point in this study was death from any cause. Secondary end points were relapse-free survival (RFS), toxicity and QOL. For RFS, an event was defined as either recurrence of cancer or a second primary tumour. Eligible patients were analysed by intention to treat.

Survival and duration of relapse-free interval was estimated with the Kaplan–Meier product limit method (Kaplan and Meier, 1958), and treatment arms were compared using the log-rank test (Peto and Peto, 1972). Treatment toxicities in the two arms were compared using the *χ*^2^ test. Changes in QOL scores from baseline were compared for the two arms using a nonparametric Mann–Whitney test.

Univariate analysis was stratified by treatment centre and performed using the log-rank test to identify characteristics predictive for survival. The prognostic factors analysed for effect were performance status, age, gender, site of primary tumour, treatment arm and tumour differentiation. Multivariate survival analysis was performed using Cox's proportional hazards model (Cox and Oakes, 1984) and corrected for all the above prognostic factors.

## RESULTS

**Figure 1:**
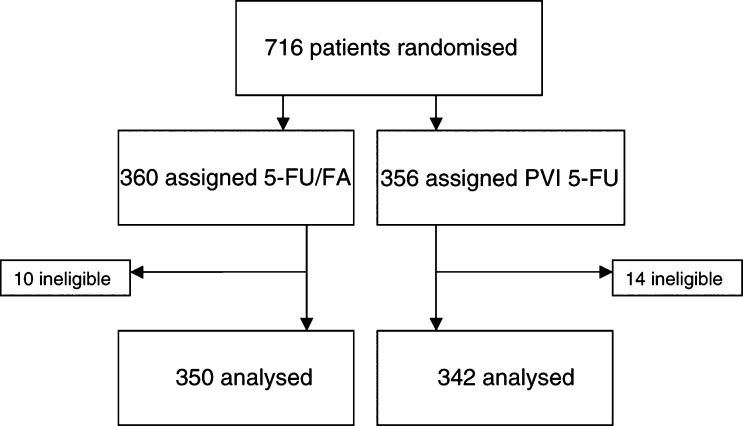
Trial profile.

In total, 716 patients with colorectal cancer from seven oncology centres in the UK were randomised. A total of 24 patients (3.4%) were ineligible mostly (20 out of 24) as a result of either unexpected metastatic disease or positive margins on review; 10 had been allocated to 5-FU/FA and 14 to PVI 5-FU (Figure 1

). The remaining 692 patients were analysed by intention to treat.

### Patient characteristics

The pretreatment characteristics of patients are listed in Table 1

**Table 1:** Characteristics of eligible patients (%)

	**5-FU/FA**		**PVI 5-FU**		**Total**	
*n*	350		342		692	
Age (years)						
Median	63		63		63	
Range	(28–95)		(27–82)		(27–95)	

Gender						
Male	181	(52)	180	(53)	361	(52)
Female	169	(48)	162	(47)	331	(48)

Site of primary tumour						
Colon	234	(67)	240	(70)	474	(68)
Rectum	116	(33)	102	(30)	218	(32)

Differentiation						
Well	11	(3)	14	(4)	25	(4)
Moderately	293	(84)	272	(80)	565	(82)
Mod-Poorly	0	(0)	1	(0.3)	1	(0.1)
Poorly	35	(10)	37	(11)	72	(10)
Unknown	11	(3)	18	(5)	29	(4)

Dukes' stage (colon)						
B	105	(45)	103	(43)	208	(44)
C	127	(54)	131	(55)	258	(54)
Unavailable	2	(1)	6	(2)	8	(1)

Dukes' stage (rectum)						
B	41	(35)	42	(41)	83	(38)
C	75	(65)	60	(59)	135	(62)

Performance status						
0	159	(45)	174	(51)	333	(48)
1	162	(46)	140	(41)	302	(44)
2	20	(6)	22	(6)	42	(6)
Unknown	9	(3)	6	(2)	15	(2)
Radiotherapy received for rectal cancer patients (*n*=218)						
Preoperative	10	(5)	4	(2)	14	(7)
Postoperative^a^	10	(5)	15	(7)	25	(12)

Figures in parentheses denote percentages of patients apart from age range.

a As part of the study protocol.

. Age, gender, performance status, Dukes' stage, differentiation and site of primary tumour were well matched between the treatment arms. Among 218 patients with rectal cancer, the operations performed were abdominal perineal resection (26%), anterior resection (69%) and others (5%). The proportion of patients undergoing each type of procedure was evenly distributed between the treatment arms. There was no difference in median time of starting chemotherapy from date of surgery – 7.7 weeks for patients in the 5-FU/FA arm *vs* 8 weeks for patients in the PVI 5-FU arm (*P*=0.143).

### Survival

**Figure 2:**
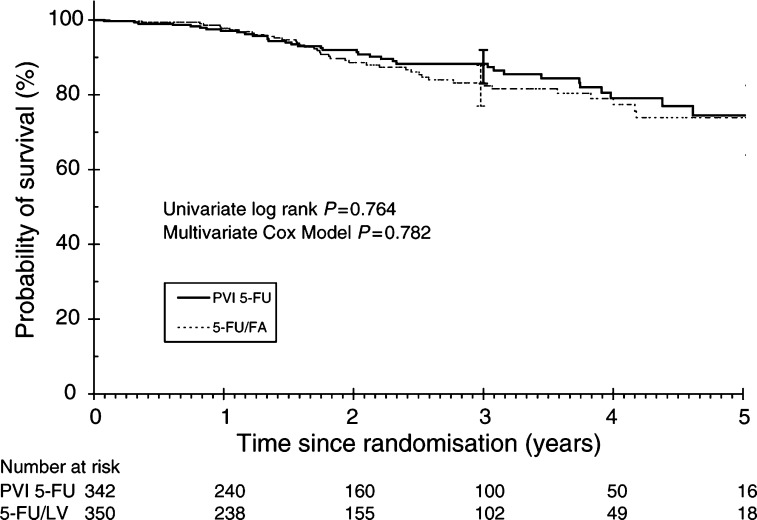
Overall survival.

This analysis was carried out at the completion of accrual, when the median duration of follow-up was 19.8 months (range 0–70) and 77 patients have died (11%). Figure 2

shows the overall survival for the two treatment arms. There was no significant differences between the two arms (log rank *P*=0.764). The 3-year survival was 83.2% (95% confidence interval (CI): 77–87.9%) for the 5-FU/FA group and 87.9% (95% CI: 83–92%) for the PVI 5-FU group. On further examination, the probability of PVI 5-FU being inferior to 5-FU/FA was only 0.0560, based on a ±5% difference in 3-year survival being ruled out. Similarly, there were no significant differences between the two treatment groups when patients were separated according to Dukes' stage (B *vs* C) or tumour sites (colon *vs* rectum). On multivariate analysis, Dukes' staging and age were significant prognostic factors whereas treatment arm was not prognostic (Table 2

**Table 2:** Multivariate analysis for overall survival

	**Variables**	***P***	**Hazard ratio**^a^	**95% CI**
All patients	Treatment arm	0.782	–	–
	Age	0.032	1.026	1.00 – 1.05
	Dukes' stage	0.004	2.052	1.25 – 3.36

Colon	Treatment arm	0.397	–	–
	Dukes' stage	0.002	2.581	1.41 – 4.74

Rectal	Treatment arm	0.08	2.254	0.91 – 5.60
	Age	0.023	1.060	1.01 – 1.12

a Hazard ratio (HR) for Dukes' stage is expressed as Dukes' C over B; hence,for all patients, Dukes' C tumours has 2.052 times higher risk of death compared to Dukes' B tumours. HR for treatment arm is expressed as 5-FU/FA over PVI 5-FU; hence in rectal cancer, 5-FU/FA arm has 2.254 times higher risk of death compared to PVI 5-FU arm.

). However for patients with rectal cancer, there was a trend towards worse survival for bolus 5-FU/FA arm (*P*=0.08).

### Relapse

**Figure 3:**
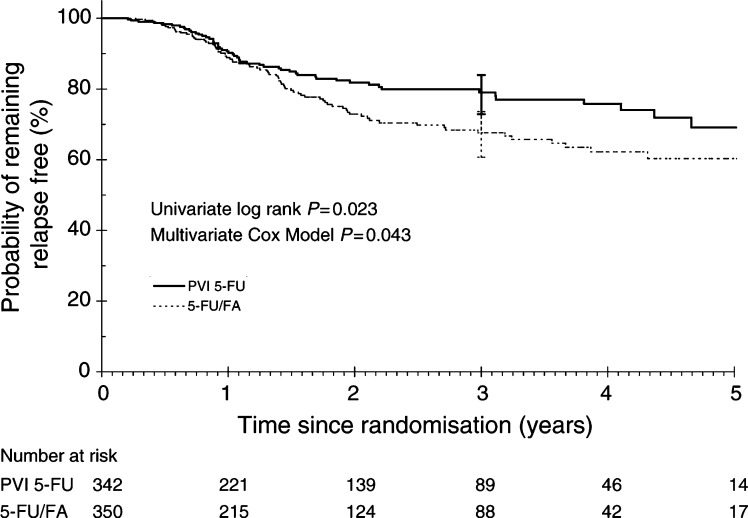
Relapse-free survival.

A total of 88 (19%) patients with colon cancer and 45 (21%) patients with rectal cancer had relapsed. Figure 3

shows the RFS for the two treatment arms. Patients treated with 5-FU/FA had significantly higher rate of relapse compared to those with PVI 5-FU (log rank *P*=0.023). The 3-year RFS was 68.3% (95% CI: 60.7–73.6%) for 5-FU/FA and 80% (95% CI: 72.9 – 83.9%) for PVI 5-FU.

Less relapses occurred in patients with Dukes' B cancer treated with PVI 5-FU (*P*=0.028), whereas no difference in relapse between the treatment arms was seen in patients with Dukes' C cancer (*P*=0.33). Relapses were significantly less in patients with rectal cancer treated with PVI 5-FU (*P*=0.007).

On multivariate analysis, treatment with 5-FU/FA was associated with worse RFS (*P*=0.043), but this effect was found to be limited to the subgroup of patients with rectal cancer. For these patients, the 5-FU/FA arm was 2.38 (95%CI: 1.27–4.45) times more at risk of relapse than those in the PVI 5-FU arm. Dukes' staging was also a significant prognostic factor for RFS (Table 3

**Table 3:** Multivariate analysis for RFS

	**Variables**	***P***	**Hazard ratio**^a^	**95% CI**
All patients	Treatment arm	0.043	1.442	1.01 – 2.06
	Dukes' stage	<0.001	2.362	1.62 – 3.45

Colon	Treatment arm	0.637	–	–
	Dukes' stage	<0.001	2.695	1.68 – 4.32

Rectal	Treatment arm	0.007	2.380	1.27 – 4.45
	Dukes' stage	0.047	1.922	1.01 – 3.66

a Hazard ratio (HR) for Dukes' stage is expressed as Dukes' C over B; hence, for all patients, Dukes' C tumours has 2.362 times higher risk of relapse compared to Dukes' B tumours. HR for treatment arm is expressed as 5-FU/FA over PVI 5-FU; hence, in rectal cancer, 5-FU/FA arm has 2.38 times higher risk of relapse compared to PVI 5-FU arm.

).

### Toxicity

There were no chemotherapy-related deaths in this study. Table 4

**Table 4:** Incidences of grades 3 and 4 toxicities

**% Worst-grade toxicity**	**5-FU/FA**	**PVI 5-FU**	***P***
Stomatitis	19.6	3.6	<0.0001
Nausea/vomiting	2.6	1.8	0.46
Diarrhoea	16.0	5.4	<0.0001
Alopecia^a^	14.3	0.3	<0.0001
Palmar-plantar syndrome	3.5	6.3	0.09
Infection	5.5	3.3	0.153
Leucopenia	10.3	0.6	<0.0001
Neutropenia	55.6	0.9	<0.0001
Thrombocytopenia	1.5	0.6	0.12
Anaemia	2.1	1.2	0.37

a Grade 2.

shows the incidences of grades 3–4 toxicities. Highly significant reduced incidences of diarrhoea, stomatitis and neutropenia occurred in patients treated with PVI 5-FU compared with 5-FU/FA. Grade 2 alopecia occurred in 25% of female patients treated with 5-FU/FA compared with 0.6% of those treated with PVI 5-FU. There was also a significant difference in the incidence of grades 1–4 nausea and vomiting (66 *vs* 25% for 5-FU/FA and PVI 5-FU groups, respectively, *P*<0.0001). Although the incidences of grades 3–4 palmar-plantar syndrome were low in both treatment arms, 72.5% of patients receiving PVI 5-FU developed ⩾grade 1 palmar-plantar syndrome compared with 47.5% of patients receiving 5-FU/FA (*P*<0.001). No difference was seen between the two treatment arms for incidence of infection or febrile episodes. For patients in the 5-FU/FA arm of the study, the number of emergency admissions was significantly greater (0.95 *vs* 1.73 in-patient bed-days, *P*=0.008) for the cohort of patients treated at Royal Marsden Hospital.

Data on adverse effects associated with Hickman lines were available for 342 of lines inserted. Serious complications were infrequent including pneumothorax (*n*=2, 0.6%) septicaemia (*n*=4, 1.2%) and thrombotic complications (*n*=25, 7%). The most frequently reported problem was superficial cutaneous infection at the line entry site, which affected 24% of Hickman lines. Other problems consisted of pain from the line entry site, shoulder, arm, back or chest, which was reported in 42 cases (12%). Only 9% of patients underwent removal and resiting of their Hickman lines.

### Chemotherapy dose intensity

The average planned dose of 5-FU per patient was 12 750 and 25 200 mg m^−2^ for the 5-FU/FA and PVI 5-FU patients, respectively. Largely, as a consequence of the dose reductions and delays arising from toxicity, patients in the bolus treatment group received on average 74% of their intended dose, while those in the PVI group received 90% of the intended dose (*P*<0.001). The proportions of patients who did not have any dose reductions, delays or interruptions during the entire treatment programme in the 5-FU/FA and PVI 5-FU groups were 13.3 and 45.7%, respectively.

### Quality of life

**Figure 4:**
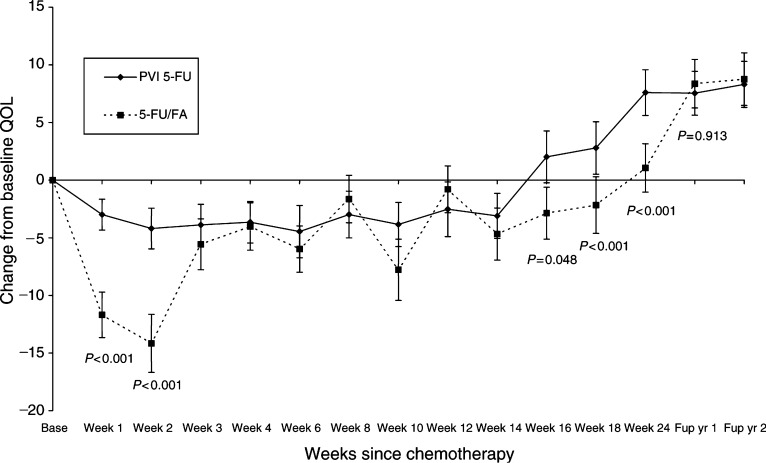
Global QOL scores (solid line PVI 5-FU, dotted line 5-FU/FA) EORTC Core 30 Global QOL scores with 95% confidence intervals. Fup yr: follow-up year.

At baseline, there was no difference in the global QOL scores between patients randomised to either 5-FU/FA or PVI 5-FU (72.0 *vs* 73.2, respectively, *P*=0.473, Figure 4

). The lowest scores for both groups were noted at the 2-week time point, but were significantly worse for patients receiving 5-FU/FA compared to those receiving PVI 5-FU (59.3 *vs* 68.6; *P*<0.001). At the 4-week time point, QOL scores for the patients in the both treatment groups equalised, coinciding with the resolution of toxicity. At 24 weeks, global scores were 74.2 and 78.8 for 5-FU/FA and PVI 5-FU, respectively (*P*<0.001), but in the follow-up period beyond 24 weeks there was no significant difference between the arms. Both arms displayed significantly increased global QOL in the follow-up period compared to the baseline.

## DISCUSSION

The data from this prospective randomised trial demonstrated the efficacy of 12 weeks' PVI 5-FU in the adjuvant setting and provide a direct comparison of the toxicity of this treatment against a standard bolus regimen. For survival there is only a 5.6% chance that PVI 5-FU is inferior to 5-FU/FA. The advantage of PVI 5-FU over 5-FU/FA is reflected by a number of parameters including toxicity and QOL. Furthermore, halving treatment duration to 12 weeks by the use of PVI 5-FU appears not to be detrimental to survival in this setting.

Previous studies designed to address the question of duration of adjuvant chemotherapy have demonstrated that 6 months of therapy provides a survival effect similar to that of 12 months (O'Connell *et al*, 1998). Survival data from the present report are derived from an analysis which was carried out when the accrual goal was reached and consequently, the duration of follow-up is relatively short with a median of only 19.8 months. However, PVI 5-FU given over 12 weeks has an effect on survival, which seems very unlikely to be inferior to that achieved by the administration of 6 months of chemotherapy with bolus 5-FU/FA. The 3-year survival of 88% in the PVI 5-FU arm corresponds to data from two other recently reported large-scale adjuvant studies in which a comparable patient population has been treated, that is, both Dukes' B and C and both colon and rectal cancers (QUASAR Collaborative Group, 2000; Taal *et al*, 2001).

When patients were analysed according to site of primary tumour, the subgroup with rectal tumours who underwent treatment with PVI 5-FU had a significantly prolonged time to relapse and a significant overall reduction in tumour recurrence compared to those receiving the standard regimen (*P*=0.043). In addition, there was a trend towards improved survival (*P*=0.08). In this group, the number of patients receiving either preoperative or postoperative radiotherapy was balanced between the two treatment arms. These results are consistent with those seen with the US Intergroup study in which patients treated with PVI 5-FU during radiation has improved RFS and overall survival compared to bolus 5-FU (O'Connell *et al*, 1994). Although FA was not used in the bolus 5-FU arm in this Intergroup study, there is no evidence to suggest combination of bolus 5-FU/FA is superior to 5-FU alone during radiation (Tepper *et al*, 2002). However, this finding in our study is based on a relatively small number of events and was the result of an unplanned subgroup analysis. To confirm this initial observation, our study has been extended for patients with rectal cancer.

Two studies comparing prolonged infusion of 5-FU with bolus regimen of 5-FU have been reported. A French study coordinated by GERCOR (Groupe d'Etude et de Recherche Clinique en Oncologie Radiotherapies) compared 2-weekly infused 5-FU/FA with bolus 5-FU/FA and a second randomisation to either 24 or 36 weeks of treatment. With 905 patients recruited, treatment was less toxic in the infused 5-FU/FA arm with significantly lower incidence of grades 3 and 4 neutropenia, diarrhoea and mucositis (*P*<0.001) (Andre *et al*, 2001). This is consistent with our study in which PVI 5-FU resulted in much better toxicity profile. No differences in disease-free survival were seen between the treatment arms or between different durations of treatment in the GERCOR study (Andre *et al*, 2002). In the Intergroup-0153 study, continuous infusion of 5-FU with levamisole was compared to bolus 5-FU/FA and levamisole. Interim analysis showed a 3-year survival of 76% for the infused arm, but the study was prematurely closed because the infused arm was unlikely to produce a survival advantage even if the accrual goal was completed (Poplin *et al*, 2000).

However, one of the critical issues in trials of adjuvant therapy for cancer patients is whether the size of the survival benefit is worth the decline in QOL caused by adverse effects of treatment. In the Intergroup-0035 study, which led to the recommendation of 1 year of 5-FU and levamisole as adjuvant therapy, 30% of patients discontinued treatment prematurely because of toxicity with a median of 5 months' treatment (Moertel *et al*, 1990). Aside from the potential impact of treatment-induced toxicity on patients' QOL, there is a detrimental impact on the actual dose of 5-FU which can be delivered. The findings of the present study showed that the higher rate of grades 3–4 toxicities experienced by patients on 5-FU/FA arm resulted in only 74% of the intended dose of 5-FU being delivered, while the favourable toxicity profile of PVI 5-FU enables patients to receive 90% of the prescribed dose.

In a previous study of patients undergoing treatment for metastatic disease, 30% of 560 catheters required premature removal because of complications (Ray *et al*, 1996). However, our results show that the most frequent complications were minor superficial infection at the line entry site, while the rate of thrombotic complications was relatively low at 7%. Only 9% of patients required a change of line. Fewer line changes in this study are likely to have arisen as a result of the lower incidence of thrombotic complications expected for patients undergoing therapy in the adjuvant setting compared to those with advanced disease. The constraints of having an indwelling line and portable electronic pumps were not associated with a negative impact on QOL, which was measured in this study by sequential QOL self-assessment. The QOL scales used detected a significant difference between the two treatment groups at the 2-week time point following initiation of treatment and favoured the PVI treatment arm. After this time point, there was no difference observed for the two groups while on treatment. However, on cessation of treatment (in the PVI 5-FU arm) at 12 weeks, a significant difference in the global QOL scores again emerged, these QOL scores improved to above baseline levels for both treatment groups after the completion of therapy, but was earlier for patients receiving PVI 5-FU.

Oral fluoropyrimidines provide prolonged 5-FU exposure at lower peak concentrations than those observed with bolus 5-FU schedule, thereby simulating continuous infusion of 5-FU. They have been shown to have similar survival to bolus 5-FU/FA in advanced disease setting and provide the same favourable toxicity profile seen with PVI 5-FU (Hoff *et al*, 2001; Van Cutsem *et al*, 2001; Carmichael *et al*, 2002; Douillard *et al*, 2002). Two trials have finished recruitment evaluating capecitabine and UFT as adjuvant therapy in colon cancer, and if their efficacy is proven in the adjuvant setting, one may be able to avoid the morbidity and inconvenience associated with central venous catheters and infusion pumps required for continuous intravenous infusion of 5-FU.

In conclusion, this study has demonstrated that the administration of infused 5-FU as adjuvant therapy is associated with less acute toxicity and less impairment of QOL than a standard bolus schedule. Furthermore, this has been achieved without any obvious adverse effect on outcome, and indeed there appears to be improved outcome, in the group of patients with rectal cancer. Reducing treatment duration to 12 weeks was not associated with any detrimental effects on the efficacy of infused 5-FU.
